# Queues with the dropping function and general service time

**DOI:** 10.1371/journal.pone.0219444

**Published:** 2019-07-17

**Authors:** Andrzej Chydzinski, Blazej Adamczyk

**Affiliations:** Institute of Informatics, Silesian University of Technology, Gliwice, Poland; Shandong University of Science and Technology, CHINA

## Abstract

We present an analysis of queueing systems with the dropping function, infinite buffer and general distribution of the service time. Firstly, a stability condition, more general than the well-known *ρ* < 1, is proven. Secondly, the formulas for the queue size distribution, loss ratio and mean duration of the busy period, are derived. Thirdly, numerical examples are given, including optimizations of the shape of the dropping function with regard to the combined cost of the queue size and loss ratio.

## 1 Introduction

A queueing system with the dropping function is a system, in which an arriving packet (job, customer) may not be allowed to the queue (dropped) with probability *d*(*n*), where *n* is the number of packets present in the queue on the new packet arrival.

Perhaps the simplest queueing system, in which some arriving packets are lost, is the commonly used system with a finite buffer and tail dropping. In such a system, an arriving packet is dropped, when the buffer is full, upon this packet arrival. Obviously, this is equivalent to the application of a trivial dropping function, in the form of the step function:

$$\begin{array}{c} d\left(n\right)=\{\begin{array}{c} 0,\;\;\;\text{if}\;\;\;n<N,\\ 1,\;\;\;\text{if}\;\;\;n\geq N, \end{array} \end{array}$$ (1)

where *N* is the buffer size.

Allowing the function *d*(*n*) to have an arbitrary, non-trivial form, can be viewed as a natural generalization of the tail-drop queueing model.

There are a few good reasons, practical and theoretical, to deal with queues with such non-trivial dropping functions.

It has been observed that the tail-drop queueing of packets in an IP network causes several problems and degrades the network performance. These include long queueing delays, synchronization of TCP flows, interflow unfairness, bursty losses (long series of losses one after another), and others. Therefore, Internet Engineering Task Force recommends in [1] the usage of active queue management (AQM), which means basically that the packets should be dropped far before the buffer gets full, or even far before the queue gets long. The dropping policy can be based on several factors, including the queue size, the loss rate, the idle periods and others. One of the most popular approaches, justified by simplicity, ease of implementation and decent performance, is the probabilistic dropping, based on non-trivial dropping functions.

Several different forms of the dropping function have been proposed for active queue management so far, starting from the simple linear function in [2], ending with the newest three-range mixture of cubic and linear functions in [3]. Other dropping functions used in active queue management include the doubly linear function [4], the exponential function [5], the quadratic function [6], orthogonal polynomials [7] and the cubic function [8]. Some AQM algorithms use dropping functions which consider additional parameters to the current queue size, e.g. the current trend of the queue’s occupancy or the packet’s flow. In the model studied herein, the dropping function is a function of the queue size only, but over a long enough time and large number of packets, the impact of the other parameters is likely to be smoothed and averaged, so the important parameter for the steady-state is the queue size. Finally, some AQM algorithms are based on a completely different, non-probabilistic approach. For an account of such algorithms we refer the reader to [9] and the references given there.

Besides networking, this study may be of some interest in economics, e.g. for the pricing of toll roads, or for defining the quality of service in call centers. In both of these examples, the buffers are virtually infinite, but a high congestion leads customers to being dropped before getting serviced. A driver may choose to drive in an alternative road, a customer may quit the call before getting a human response or may be rejected by the system upon calling, when the queue is long. Using the results presented here, it is possible to control and optimize the customer loss process, rather than leaving it to happen spontaneously. For instance, by rejecting a place in the queue to some customers when the queue is long, we may achieve a good trade-off between the average waiting time and the customer loss ratio.

Therefore, in this paper we deal with the classic M/G/1 queueing model with the addition of a dropping mechanism based on a non-trivial dropping function. The importance of the M/G/1 system can be seen in virtually all queueing theory textbooks—this is one of the most frequently solved and discussed models. From the networking perspective, the usefulness of this model is connected with the fact, that it allows an arbitrary distribution of the service time. In most TCP/IP networks, the packet size (and the resulting service time) varies significantly, but not according to the exponential distribution—most of the probability mass is concentrated on the maximal and minimal size (e.g. 40 and 1500 bytes).

We begin the analysis with formulating and proving a sufficient condition for the system stability. This is an absolutely necessary step in an analysis of all systems, in which the queue can potentially grow to infinity. As pointed out in earlier works, the classic stability condition, *ρ* < 1, is far too restrictive, when dealing with the systems with the dropping function. It is easy to find models with dropping functions which are stable even when *ρ* is greater than one. Consider, for instance, a simple system with *ρ* = 5 and *d*(*n*) = 0.9 for every *n*. For the M/M/1 model with the dropping function, several stability conditions were given in [10]. However, the model of [10] assumes that the queue size process is Markovian. Herein, the queue size process is not Markovian, so finding and proving a stability condition requires a totally different approach. On the other hand, the general service time is necessary in modeling of TCP/IP networks, as the packet processing time is far from exponential.

Then the formulas for three important characteristics—the queue size distribution, the mean busy period and the loss ratio—are derived. The distribution of the queue size is the first, most important characteristic in an analysis of every queueing system, usually accompanied by an analysis of the busy period. The loss ratio, *L*, defined as the long-run fraction of dropped packets, is computed only for systems with losses, as the one considered herein. Obviously, the loss ratio is equivalent to the loss probability—both terms can be interchanged. The loss ratio is especially important in networking, where the fraction of lost packets has a deep impact on the communication quality. Therefore, it has been studied extensively from networking perspective using measurements [11–15] and mathematical modeling [16–18]. Besides the loss ratio, the statistical structure of packet losses has been investigated using theoretical and experimental tools in [19, 20].

We give also several numerical examples. Firstly, a cost function combining the queue size and the loss ratio is proposed. Then, it is minimized using a class of dropping functions, depending on a parameter, thus providing a good trade-off between the two contradicting goals—a short queue and a small loss ratio. Then, the dependence of the system performance on the standard deviation of the service time, and on the system load, is studied.

The rest of the paper is organized as follows. In Section 2, the previous work on queues with dropping functions and queues with state-dependent rates is recalled and characterized briefly. In Section 3, the queueing model and notation is introduced. Then, in Section 4, the stability condition is proven. In Section 5, the most important performance characteristics are shown, including the queue size distribution, the loss ratio and the average duration of the busy period. In Section 6, numerical results motivated by a need for a dropping function that balances the average queue size and the loss ratio, are presented first. Then the impact of the standard deviation of the service time and the system load is tested. Finally, conclusions are gathered in Section 7.

## 2 Related work

We are unaware of any previous work studying the combination of an M/G/1 queue with an infinite buffer and a non-trivial dropping function.

As far as we know, there is only one analytical article [10], on the queueing model with the dropping function and infinite buffer. But the service time, as well as the interarrival time, are both exponential in [10]. As mentioned above, these make the model of [10] to be fully Markovian, with the birth-and-death process structure, and allows for application of several well-known analytical tools. Herein, the situation is different. Assuming the general type of the service time distribution ruins the Markovian structure and makes all derivations much more difficult. For instance, the main stability theorem requires applying advanced results of the renewal theory and computing some special characteristics of the M/G/1 queue, which is not needed in the proof of Theorem 1 of [10].

The queueing literature on models with the dropping function, but a finite buffer, is much wider. The studies began with an approximate solution of the queue with batch Poisson arrivals and a linear dropping function in [21]. Then, an approximate solution of the system with general batch arrivals and exponential service time was obtained in [22]. The exact solution of the system with Poisson arrivals and exponential service time was found in [23]. The stationary solution of the model with the general service time was shown in [24]. In [25], the transient solution of the same model was derived, while in [26], a case with multiple service stations was analyzed. In [27, 28], queues with a more complex arrival structure were analyzed, including the autocorrelation of the interarrival times in [27], and the full structure of the batch Markovian arrival process in [28]. Finally, in [29] a generalization of the queueing system with the dropping function was proposed and analyzed. This generalization assumes continuous job sizes and buffer size.

The model studied herein is somewhat similar to the model with state-dependent rates, see [30–32] and the references given there. There are, however, several differences between the two models. These differences are fundamental and make it impossible to translate one model to the other. Namely, in [30–32], the arrival and service rates depend on the system workload, i.e. the total time needed to service the whole queue. What is important, the service time of a new customer becomes known immediately upon arrival of this customer, so the system workload is always known. For this reason, the arrival rate can be changed to adopt to the current workload. We, however, assume no knowledge about the current system workload. The service time of a customer becomes known upon departure of this customer, not upon arrival. The only thing always known herein is the queue size. Therefore, the arrival rate depends on the queue size, not the unknown workload. Moreover, we assume that the arrival rate cannot be controlled directly, so the dropping function is used for thinning the arrival process, at the cost of losses. There are no losses in the model of [30–32], except perhaps for those caused by a finite buffer, if assumed (both variants are analyzed, with a finite and infinite buffer). Finally, the assumption of knowing the current system workload might not be practical in some applications of the queueing system. In the mentioned call center example, it is obviously impossible to know in advance how long it will take to service the current queue. In a TCP/IP network node, it is also hard to predict in advance the total packet service time (see [33] for more details). For instance, all the packets of one flow may need forwarding only, while all the packets of another flow may first require a deep packet inspection, IPSec processing and, finally, forwarding. Also, the first and the last packet in a flow may need a longer processing time, as the processor has to open or close connection states. Prediction of the total packet service time is possible in theory, but requires a lot of computations, which make it impractical.

In the proof of the stability condition (Section 4), we exploit an approach based on the renewal theory in general, and the key renewal theorem in particular. This approach has proven to be useful in finding the limiting distributions in classic queues, like in [34], more complex queues, like in [35], and in other engineering problems, like in [36].

## 3 Description of the model

We deal with a single-server queueing model. The arrival stream is Poisson with rate λ. The service time is distributed according to distribution function *F*(⋅), which is not further specified.

The buffer (waiting room) is infinite.

Additionally, a dropping mechanism based on the dropping function *d*(⋅) is applied, which means that every arriving packet may be dropped with probability *d*(*n*), where *n* is the queue size at time of this packet arrival, including the packet being serviced, if applicable. The dropping function *d*(*n*) is not further specified and may assume any value in interval [0, 1) for every *n* = 0, 1, 2, …. We exclude the possibility that *d*(*n*) = 1 for some *n*, as it is equivalent to having a finite, rather than infinite, buffer.

The queueing discipline is irrelevant to this study, it can be FIFO, LIFO, etc.

The load offered to the queue (or simply, load) is defined as:

$$\begin{array}{c} \rho =\lambda m, \end{array}$$ (2)

where

$$\begin{array}{c} m=\int_{0}^{\infty }tdF\left(t\right) \end{array}$$ (3)

is the average service time.

Let *X*(*t*) denote the queue size at time *t*, including the service position, if occupied. If not stated otherwise, it is assumed that at *t* = 0 the system is empty, i.e. *X*(0) = 0. Let P denote probability and E—the average value of a random variable.

## 4 Stability of the system

We consider a queueing system to be stable if and only if for every *n* = 0, 1, …, there exist the limit:

$$\begin{array}{c} \lim_{t\to \infty }P\left(X\left(t\right)=n\right)=p_{n}, \end{array}$$ (4)

and it holds:

$$\begin{array}{c} \sum_{n=0}^{\infty }p_{n}=1. \end{array}$$ (5)

If the limit does not exist for some *n*, or the sum in (5) is not equal to 1, we consider the system to be unstable.

A single-server queue with an infinite buffer is stable if *ρ* < 1 [34, 37]. When the dropping function is applied, this assumption becomes excessive. Obviously, if *ρ* < 1, then the system with the dropping function is stable—it would have been stable even without dropping of packets. But following the example given in the introduction, we can easily construct stable systems with the dropping function and arbitrary large *ρ*, for instance 1000.

The following theorem provides a tighter stability condition.

**Theorem 1**. *If*

$$\begin{array}{c} m<\infty , \end{array}$$ (6)

*and*

$$\begin{array}{c} c=\underset{n\to \infty }{lim sup}\rho \left(1-d\left(n\right)\right)<1, \end{array}$$ (7)

*then the M/G/1 queue with the dropping function d*(*n*) *is stable*.

Proof. The proof will be divided into several distinct conceptual parts (*Part 1, Part 2*, etc.), starting with an outline, which gives a high-level overview of the proof.

*Outline*. In *Part 1*, a simple dropping function, *d*_0_, which is smaller or equal to *d* for every *n*, is proposed. This function has a form of the step function and assumes only two values. As this function drops less packets than *d* for every possible queue size, the stability of the system under study follows from the stability of the system with *d*_0_ instead of *d*, and other parameters unaltered. Therefore, in *Part 2* the stability of the system with *d*_0_ is proven. This is the longest part of the proof and is divided into several pieces (*Part 2a, Part 2b*, etc.). In *Part 2a*, the key renewal theorem is used to obtain the limiting distribution of the queue size in the system with *d*_0_. It has the form $q_{n}=\frac{K_{n}+L_{n}}{C}$, where *K*_*n*_ and *L*_*n*_ are some special characteristics of the classic M/G/1 queue, while *C* is a special characteristic of the system with the dropping function *d*_0_. The rest of *Part 2* is devoted to show, that *K*_*n*_, *L*_*n*_ and *C* are finite, given the assumptions of Theorem 1, and that the resulting limiting distribution *q*_*n*_ is not defective, i.e. $\sum_{n=0}^{\infty }q_{n}=1$. In *Part 2b*, using some known results on the M/G/1 queue, it is shown that *K*_*n*_ is finite. Similarly, in *Part 2c* it is shown that *L*_*n*_ is finite. In *Part 2d* it is proven that *C* is finite and $\sum_{n=0}^{\infty }q_{n}=1$ holds. Finally, *Part 3* concludes the whole proof by gathering all partial results together.

*Part 1*. Let us first find a new, simpler dropping function *d*_0_, which is smaller or equal to *d* for every *n* and such that it makes the system stable.

If (7) is fulfilled, then there must exist natural number *n*_0_ such that:

$$\begin{array}{c} \rho \left(1-d\left(n\right)\right)<\frac{c+1}{2}<1,\;\;\;\;n>n_{0}. \end{array}$$ (8)

Let us define the new dropping function in the following way:

$$\begin{array}{c} d_{0}\left(n\right)=\{\begin{array}{c} 1-\frac{c+1}{2\rho },\;\;\;\text{if}\;\;\;n>n_{0},\\ 0,\;\;\;\text{otherwise}. \end{array} \end{array}$$ (9)

From (8) it follows that:

$$\begin{array}{c} d_{0}\left(n\right)\leq d\left(n\right),\;\;\;\;\;\;n=0,1,2,\ldots . \end{array}$$ (10)

Now we have to show that the new queueing system, with the dropping function *d*_0_ and other parameters unaltered, is stable. We will denote by *X*_0_(*t*) the queue size at time *t* in the new system. This will make it possible to distinguish it from the queue size in the original system, denoted by *X*(*t*).

*Part 2a*. Let us first define the following sequence of moments in time:

$$\begin{array}{c} 0=t_{0}<t_{1}<t_{2}<t_{3}<\ldots , \end{array}$$

as the consecutive moments of reaching by the queue size levels *n*_0_ and *n*_0_ + 1, alternately. Namely, if *X*_0_(0) ≤ *n*_0_, then *t*_1_ is the first time when the queue size reaches *n*_0_ + 1, then *t*_2_ is the first time after *t*_1_ when the queue size reaches *n*_0_, then *t*_3_ is the first time after *t*_2_ when the queue size reaches *n*_0_ + 1, and so on. If *X*_0_(0) > *n*_0_, then *t*_1_ is the first time when the queue size reaches *n*_0_, then *t*_2_ is the first time after *t*_1_ when the queue size reaches *n*_0_ + 1, then *t*_3_ is the first time after *t*_2_ when the queue size reaches *n*_0_, and so on.

Formally, if *X*(0) ≤ *n*_0_, then *t*_*k*_’s are defined as:

$$\begin{array}{c} t_{1}=\inf\left\{t>0:X\left(t\right)=n_{0}+1\right\}, \end{array}$$

$$\begin{array}{c} t_{2}=\inf\left\{t>t_{1}:X\left(t\right)=n_{0}\right\}, \end{array}$$

$$\begin{array}{c} t_{3}=\inf\left\{t>t_{2}:X\left(t\right)=n_{0}+1\right\}, \end{array}$$

and so on. If *X*(0) > *n*_0_, then *t*_*k*_’s are defined as:

$$\begin{array}{c} t_{1}=\inf\left\{t>0:X\left(t\right)=n_{0}\right\}, \end{array}$$

$$\begin{array}{c} t_{2}=\inf\left\{t>t_{1}:X\left(t\right)=n_{0}+1\right\}, \end{array}$$

$$\begin{array}{c} t_{3}=\inf\left\{t>t_{2}:X\left(t\right)=n_{0}\right\}, \end{array}$$

and so on.

Using the notation:

$$\begin{array}{c} G\left(x\right)=P(t_{2}<x|X_{0}\left(0\right)=n_{0}), \end{array}$$

$$\begin{array}{c} G^{0*}\left(u\right)=1,\;\;\;\;\;\;G^{k*}\left(u\right)=\int_{0}^{u}G^{(k-1)*}\left(u-v\right)dG\left(v\right),\;\;\;\;k\geq 1, \end{array}$$ (11)

and the renewal function:

$$\begin{array}{c} M\left(u\right)=\sum_{k=0}^{\infty }G^{k*}\left(u\right), \end{array}$$ (12)

we can derive the distribution of the queue size in the new system at arbitrary time. We have:

$$\begin{array}{cc} P(X_{0}\left(t\right) & =l|X_{0}\left(0\right)=n_{0})=\sum_{k=0}^{\infty }P\left(X_{0}\left(t\right)=l,t_{2k}\leq t<t_{2k+2}|X_{0}\left(0\right)=n_{0}\right)\\ & =\sum_{k=0}^{\infty }\int_{0}^{t}P\left(X_{0}\left(t\right)=l,t_{2k}\leq t<t_{2k+2}|t_{2k}=u,X_{0}\left(0\right)=n_{0}\right)dG^{k*}\left(u\right)\\ & =\sum_{k=0}^{\infty }\int_{0}^{t}P\left(X_{0}\left(t-u\right)=l,t_{2}>t-u|X_{0}\left(0\right)=n_{0}\right)dG^{k*}\left(u\right)\\ & =\int_{0}^{t}P\left(X_{0}\left(t-u\right)=l,t_{2}>t-u|X_{0}\left(0\right)=n_{0}\right)dM\left(u\right). \end{array}$$

Using the latter formula and the key renewal theorem [34], p. 102, yields:

$$\begin{array}{c} q_{l}=\lim_{t\to \infty }P\left(X_{0}\left(t\right)=l\right)=\frac{1}{C}\int_{0}^{\infty }P\left(X_{0}\left(t\right)=l,t_{2}>t|X_{0}\left(0\right)=n_{0}\right)dt,\;\;\;\;l\geq 0, \end{array}$$ (13)

where *C* is the expected value of *t*_2_, if the system starts from level *n*_0_, i.e.:

$$\begin{array}{c} C=E(t_{2}|X_{0}\left(0\right)=n_{0}). \end{array}$$ (14)

From (13) it follows then:

$$\begin{array}{cc} q_{l} & =\frac{1}{C}\int_{0}^{\infty }P\left(X_{0}\left(t\right)=l,t_{1}>t,t_{2}>t|X_{0}\left(0\right)=n_{0}\right)dt\\ & \;+\frac{1}{C}\int_{0}^{\infty }P\left(X_{0}\left(t\right)=l,t_{1}\leq t,t_{2}>t|X_{0}\left(0\right)=n_{0}\right)dt\\ & =\frac{1}{C}\int_{0}^{\infty }P\left(X_{0}\left(t\right)=l,t_{1}>t|X_{0}\left(0\right)=n_{0}\right)dt\\ & \;+\frac{1}{C}\int_{0}^{\infty }dt\int_{0}^{t}P\left(X_{0}\left(t\right)=l,t_{2}>t|t_{1}=u,X_{0}\left(0\right)=n_{0}\right)dP\left(t_{1}<u|X_{0}\left(0\right)=n_{0}\right)\\ & =\frac{1}{C}\int_{0}^{\infty }P\left(X_{0}\left(t\right)=l,t_{1}>t|X_{0}\left(0\right)=n_{0}\right)dt\\ & \;+\frac{1}{C}\int_{0}^{\infty }P\left(X_{0}\left(t\right)=l,t_{1}>t|X_{0}\left(0\right)=n_{0}+1\right)dt, \end{array}$$

which gives the final result of this part, namely:

$$\begin{array}{c} q_{l}=\frac{K_{l}+L_{l}}{C}, \end{array}$$ (15)

with

$$\begin{array}{c} K_{l}=\int_{0}^{\infty }P\left(X_{0}\left(t\right)=l,t_{1}>t|X_{0}\left(0\right)=n_{0}\right)dt, \end{array}$$

$$\begin{array}{c} L_{l}=\int_{0}^{\infty }P\left(X_{0}\left(t\right)=l,t_{1}>t|X_{0}\left(0\right)=n_{0}+1\right)d, \end{array}$$

and *C* defined in (14).

To prove the stability of the system with the dropping function *d*_0_, it is sufficient to show that *C*, *K*_*l*_ and *L*_*l*_ are finite, and that:

$$\begin{array}{c} \sum_{l=0}^{\infty }q_{l}=1. \end{array}$$ (16)

*Part 2b*. It is easily seen that the system with *d*_0_ operates in the same way as the classic M/G/1 queue, except for the fact, that the effective arrival rate is λ when the queue size is less or equal to *n*_0_, and $\mu =\lambda \frac{c+1}{2\rho }$, when the queue size is greater than *n*_0_. Moreover, *K*_*l*_ and *L*_*l*_ are defined for one, unaltered arrival rate only—the former for λ, the latter for *μ*. Therefore, *K*_*l*_ and *L*_*l*_ constitute some special characteristics of the classic M/G/1 queueing system. Fortunately, derivations of both of these characteristics of the M/G/1 queue were already done and can be found in literature.

To study characteristic *K*_*l*_, which is a characteristic of the classic M/G/1 queue with arrival rate λ, the results of [35] can be used. Namely, under assumption (6), adapting the notation and repeating the derivations of Section 4 of [35] give:

$$\begin{array}{c} K_{l}=\frac{\sum_{k=1}^{n_{0}}d_{l-n_{0}-1+k}\left(R_{n_{0}+1-k}-R_{n_{0}-k}\right)+\frac{I(l=0)}{\lambda }}{\sum_{k=0}^{n_{0}}r_{k}\left(R_{n_{0}+1-k}-R_{n_{0}-k}\right)}<\infty , \end{array}$$ (17)

where

$$\begin{array}{c} r_{k}=\int_{0}^{\infty }\frac{e^{-\lambda t}{\left(\lambda t\right)}^{k}}{k!}dF\left(t\right), \end{array}$$ (18)

$$\begin{array}{c} d_{k}=I\left(k\geq 0\right)\int_{0}^{\infty }\frac{e^{-\lambda t}{\left(\lambda t\right)}^{k}}{k!}\left(1-F\left(t\right)\right)dt, \end{array}$$ (19)

$$\begin{array}{c} R_{0}=0,\;\;\;\;R_{1}=\frac{1}{r_{0}},\;\;\;\;\;\;\;\;R_{k+1}=R_{1}\left(R_{k}-\sum_{i=0}^{k}r_{i+1}R_{k-i}\right),\;\;\;k\geq 1, \end{array}$$ (20)

and *I* is an indicator function defined as: *I*(*A*) = 1 if *A* is fulfilled, *I*(*A*) = 0 otherwise.

*Part 2c*. To obtain *L*_*l*_, the results of the same paper can be used, but another assumption, besides (6), is required for this:

$$\begin{array}{c} \rho^{\prime }=m\mu <1. \end{array}$$ (21)

This assumption is fulfilled, as we have:

$$\begin{array}{c} m\mu =m\lambda \frac{c+1}{2\rho }=\frac{c+1}{2}<1. \end{array}$$ (22)

Therefore, adapting the notation and repeating the derivations of Section 6 of [35] yields:

$$\begin{array}{cc} L_{l} & =\{\begin{array}{c} \varphi_{l}[\frac{Q_{l-n_{0}}}{Q_{1}}\;+\;\sum_{k=1}^{l-n_{0}}\left(\sum_{j=0}^{k}b_{j}-1\right)Q_{l-n_{0}-k}]\;-\;\sum_{k=1}^{l-n_{0}}f_{k-1}Q_{l-n_{0}-k},\;\;\text{if}\;l>n_{0}\;+\;1,\\ \varphi_{l},\;\;\;\;\;\;\;\text{if}\;\;\;\;\;\;\;l=n_{0}+1,\\ 0,\;\;\;\;\;\;\;\text{otherwise,}\;\;\; \end{array}\\ & <\infty , \end{array}$$ (23)

where

$$\begin{array}{c} \varphi_{l}=\frac{\sum_{k=1}^{l-n_{0}+1}f_{k-1}Q_{l-n_{0}+1-k}}{Q_{l-n_{0}+1}/Q_{1}+\sum_{k=1}^{l-n_{0}+1}\left(\sum_{j=0}^{k}b_{j}-1\right)Q_{l-n_{0}+1-k}}, \end{array}$$ (24)

$$\begin{array}{c} b_{k}=\int_{0}^{\infty }\frac{e^{-\mu t}{\left(\mu t\right)}^{k}}{k!}dF\left(t\right), \end{array}$$ (25)

$$\begin{array}{c} f_{k}=I\left(k\geq 0\right)\int_{0}^{\infty }\frac{e^{-\mu t}{\left(\mu t\right)}^{k}}{k!}\left(1-F\left(t\right)\right)dt, \end{array}$$ (26)

$$\begin{array}{c} Q_{0}=0,\;\;\;\;Q_{1}=\frac{1}{b_{0}},\;\;\;\;\;\;\;\;Q_{k+1}=Q_{1}\left(Q_{k}-\sum_{i=0}^{k}b_{i+1}Q_{k-i}\right),\;\;\;k\geq 1. \end{array}$$ (27)

*Part 2d*. To compute *C*, it can be observed that it is equal to the sum of the average time that takes the queue size to reach level *n*_0_ + 1 from level *n*_0_, plus the average time back, from level *n*_0_ + 1 to *n*_0_. Writing this formally we get:

$$\begin{array}{c} C=E\left(t_{2}|X_{0}\left(0\right)=n_{0}\right)=E\left(t_{1}|X_{0}\left(0\right)=n_{0}\right)+E\left(t_{1}|X_{0}\left(0\right)=n_{0}+1\right). \end{array}$$ (28)

Then we obtain:

$$\begin{array}{c} E\left(t_{1}|X_{0}\left(0\right)=n_{0}\right)=\int_{0}^{\infty }P\left(t_{1}>t|X_{0}\left(0\right)=n_{0}\right)dt=\sum_{l=0}^{n_{0}}K_{l}, \end{array}$$ (29)

which is finite, as a finite sum of finite summands. As for the second summand of (28), we have:

$$\begin{array}{c} E\left(t_{1}|X_{0}\left(0\right)=n_{0}+1\right)=\int_{0}^{\infty }P\left(t_{1}>t|X_{0}\left(0\right)=n_{0}+1\right)dt=\sum_{l=n_{0}+1}^{\infty }L_{l}. \end{array}$$ (30)

In Section 6 of [35], the latter sum was proven to be finite under assumption (21) and obtained in the following simple form:

$$\begin{array}{c} E\left(t_{1}|X_{0}\left(0\right)=n_{0}+1\right)=\frac{m}{1-m\mu }. \end{array}$$ (31)

Thus we can conclude that:

$$\begin{array}{c} C=E\left(t_{2}|X_{0}\left(0\right)=n_{0}\right)=\sum_{l=0}^{n_{0}}K_{l}+\frac{m}{1-m\mu }<\infty . \end{array}$$ (32)

Therefore, it suffices to show that (16) is fulfilled. From (15), (28), (29) and (30) it follows that:

$$\begin{array}{c} \sum_{l=0}^{\infty }q_{l}=\frac{1}{C}\sum_{l=0}^{\infty }\left(K_{l}+L_{l}\right)=\frac{1}{\sum_{l=0}^{n_{0}}K_{l}+\sum_{l=n_{0}+1}^{\infty }L_{l}}\sum_{l=0}^{\infty }\left(K_{l}+L_{l}\right)=1, \end{array}$$ (33)

The latter equality follows from the fact that *K*_*l*_ = 0 for *l* > *n*_0_ and *L*_*l*_ = 0 for 0 ≤ *l* ≤ *n*_0_.

*Part 3*. Summarizing, in (9) a simple dropping function *d*_0_, smaller or equal to *d* was proposed. It was shown that the limiting queue size distribution for *d*_0_ has form (15). In (17), (23) and (32) it was proven that all parts of (15) are finite, given the assumptions of the theorem. Then, in (33) it was shown that the limiting distribution of the queue size for *d*_0_ is not defective. This completes the proof of the stability of the system with the dropping function *d*_0_. Finally, using (10) completes the proof of Theorem 1.

## 5 Queue size, loss ratio and busy period

Before the stationary distribution of the queue size, *p*_*n*_, can be computed, we have to introduce some notation. Namely, let the queueing system be stable and *X*_*n*_, *n* = 1, 2, …, denote the queue size just after the *n*-th departure time. We define *π*_*k*_ as:

$$\begin{array}{c} \pi_{k}=\lim_{n\to \infty }P\left(X_{n}=k\right),\;\;\;\;k=0,1,\ldots . \end{array}$$ (34)

We will derive first formulas for the stationary distribution of the queue size and the loss ratio as functions of *π*_*k*_. Then it will be shown, how *π*_*k*_ values can be computed.

Let *L* denote the loss ratio, i.e. the long-run fraction of dropped packets.

**Theorem 2**. *If the M/G/1 system with the dropping function d*(⋅) *is stable then its loss ratio equals*:

$$\begin{array}{c} L=1-\frac{1}{\rho }+\frac{\pi_{0}}{\rho^{2}\left(1-d\left(0\right)\right)+\rho \pi_{0}}, \end{array}$$ (35)

*while the stationary distribution of the queue size equals*:

$$\begin{array}{c} p_{n}=\left(1-L\right)\frac{\pi_{n}}{1-d(n)},\;\;\;\;n=0,1,\ldots . \end{array}$$ (36)

Proof. Firstly, we apply Burke’s theorem from p. 7 of [38]. It states that for a stochastic process, whose all trajectories are step functions with unit jumps, the stationary distribution for moments just after the step down must be equal to the stationary distribution for moments just before the step up, if either of the distributions exists.

Namely, let ${\hat{t}}_{1},{\hat{t}}_{2},{\hat{t}}_{3},\ldots$ denote consecutive moments, when a packet is accepted to the queue, and let ${\hat{p}}_{k}$ denote the stationary distribution of the queue size just before the packet acceptance, i.e.:

$$\begin{array}{c} {\hat{p}}_{n}=\lim_{k\to \infty }P\left(X\left({\hat{t}}_{k}-\right)=n\right),\;\;\;\;n=0,1,\ldots . \end{array}$$ (37)

Applying Burke’s theorem yields then:

$$\begin{array}{c} {\hat{p}}_{n}=\pi_{n},\;\;\;\;n=0,1,\ldots . \end{array}$$ (38)

Secondly, recalling the PASTA property of the Poisson process, [39], it can be concluded that the stationary probability of the queue size *n* at the packet arrival epoch is equal to *p*_*n*_ for every *n* = 0, 1, …. The only assumption of PASTA is fulfilled here in an obvious way—the future increments of the Poisson process after time *t* do not depend on the queue size before *t*.

The latter conclusion can be used then to establish the relation between ${\hat{p}}_{n}$ and *p*_*n*_. Let *t*_*a*_ denote an arbitrary Poisson arrival time and let *A*(*t*_*a*_) denote the event that the packet arriving at time *t*_*a*_ is accepted. From the definition of *L* we have $P(A\left(t_{a}\right))=1-L$, while the definition of ${\hat{p}}_{n}$ yields ${\hat{p}}_{n}=P\left(X\left(t_{a}\right)=n|A\left(t_{a}\right)\right)$. Thus we have:

$$\begin{array}{c} {\hat{p}}_{n}=P\left(X\left(t_{a}\right)=n|A\left(t_{a}\right)\right)=\frac{P(X\left(t_{a}\right)=n,A\left(t_{a}\right))}{P(A\left(t_{a}\right))}=\frac{p_{n}\left(1-d\left(n\right)\right)}{1-L},\;\;\;\;n=0,1,\ldots . \end{array}$$ (39)

In the last step, the fact that probability of the queue size *n* at the packet arrival epoch is *p*_*n*_ was used.

Now, combining (39) with (38) and solving the resulting equation with respect to *p*_*n*_, we can finish the proof of (36). Note that the assumption *d*(*n*) < 1 is needed to derive *p*_*n*_ from (39) and (38).

To compute the loss ratio, we can use a formula valid for general class of single-server queueing systems with losses, which can be found for instance in [28], namely:

$$\begin{array}{c} L=1-\frac{1-p_{0}}{\rho }, \end{array}$$ (40)

where *p*_0_ is the stationary empty queue probability. Substituting *n* = 0 to (36) gives:

$$\begin{array}{c} p_{0}=\left(1-L\right)\frac{\pi_{0}}{1-d(0)}. \end{array}$$ (41)

Combining (40) with (41) yields:

$$\begin{array}{c} p_{0}=\frac{\pi_{0}}{\rho \left(1-d\left(0\right)\right)+\pi_{0}}. \end{array}$$ (42)

Finally, substituting (42) to (40) we obtain (35), which completes the proof of the theorem.

In Theorem 2 it was assumed that *d*(*n*) < 1 for every *n*. If the dropping function is 1 for some *n*_0_, then the system is in fact equivalent to the system with a finite buffer of size *n*_0_. The distribution of the queue size and the loss ratio for the finite-buffer M/G/1/N system were obtained in [24].

To use effectively the proven formulas, the vector:

$$\begin{array}{c} \pi =(\pi_{0},\pi_{1},\pi_{2},\ldots ) \end{array}$$ (43)

has to be first computed. If the queueing system is stable, then the sequence *X*_*n*_ constitutes an ergodic Markov chain, with transition probabilities:

$$\begin{array}{c} p_{j,k}=P\left(X_{n+1}=k|X_{n}=j\right),\;\;\;\;\;j,k=0,1,2,\ldots , \end{array}$$ (44)

and transition matrix:

$$\begin{array}{c} P={\left[p_{j,k}\right]}_{j,k=0,1,2,\ldots }. \end{array}$$ (45)

Therefore *π*, which is the stationary vector for *P*, has to fulfill the standard system of equations:

$$\begin{array}{c} \pi P=\pi , \end{array}$$ (46)

and

$$\begin{array}{c} \sum_{j=0}^{\infty }\pi_{j}=1. \end{array}$$ (47)

This system is infinite, but this is not a problem. In [40] it was shown, that the solution _(*n*)_*π* of the truncated system:

$$\begin{array}{c} {}_{\left(n\right)}\pi {}_{\left(n\right)}P={}_{\left(n\right)}\pi , \end{array}$$ (48)

converges elementwise to *π* as *n* → ∞, where _(*n*)_*P* is the *n* × *n* northwest corner truncation of the matrix *P*. Therefore, the stationary vector *π* can be obtained with a high precision by solving a finite linear system of equations.

What is left, is to show how transition probabilities *p*_*j*,*k*_ can be computed up to some arbitrary large *j* and *k*. This problem has already been solved in [24] in the case of the finite-buffer system with the dropping function. This solution is applicable here as well. Firstly, let us denote by *Q*_*n*,*k*_(*u*) the probability, that in interval (0, *u*], *k* packets are accepted to the queue, provided that *X*(0) = *n* and there is no service completion by time *u*. Using *Q*_*n*,*k*_(*u*), we can compute probability *a*_*n*,*k*_ that *k* packets are accepted to the queue during the service time, provided that *X*(0) = *n*. Namely, we have:

$$\begin{array}{c} a_{n,k}=\int_{0}^{\infty }Q_{n,k}\left(u\right)dF\left(u\right). \end{array}$$ (49)

Using *a*_*n*,*k*_ values, transition probabilities:

$$\begin{array}{c} p_{j,k}=\{\begin{array}{ccc} a_{1,k}, & \text{if} & j=0,k\geq 0,\\ a_{j,k-j+1}, & \text{if} & j\geq 1,k\geq j-1,\\ 0, & & \text{otherwise}, \end{array} \end{array}$$ (50)

can be easily obtained. Finally, *Q*_*n*,*k*_(*u*) can be computed from its Laplace transform:

$$\begin{array}{c} q_{n,k}\left(s\right)=\int_{0}^{\infty }e^{-su}Q_{n,k}\left(u\right)du, \end{array}$$ (51)

which in [24] was proven to be equal:

$$\begin{array}{c} q_{n,k}\left(s\right)=\frac{\prod_{i=0}^{k-1}\lambda \left(1-d\left(n+i\right)\right)}{\prod_{i=0}^{k}\left(s+\lambda -\lambda d\left(n+i\right)\right)},\;\;\;\;\;\;\;n\geq 0,\;\;k\geq 0. \end{array}$$ (52)

In numerical calculations, (52) has to be inverted using an inversion method for the Laplace transform. Many such methods are available. We used the Zakian method of [41] to obtain results presented in Section 6.

We finish derivations of the most important characteristics of the model with the busy period.

**Theorem 3**. *If the M/G/1 system with the dropping function is stable, then its average duration of the busy period is*:

$$\begin{array}{c} Eb=\frac{\rho }{\lambda \pi_{0}}. \end{array}$$ (53)

Proof. It is easily seen that an empty system remains empty for an exponentially distributed time with parameter (1 − *d*(0))λ. Therefore, the expected duration of the idle period of the system is equal to:

$$\begin{array}{c} \frac{1}{\lambda (1-d(0))}. \end{array}$$ (54)

On the other hand, the expected duration of the busy period, divided by the expected duration of the idle period must be the same, as the non-empty system probability, divided by the empty system probability. This yields:

$$\begin{array}{c} \frac{Eb}{\;\frac{1}{\lambda (1-d(0))}\;}=\frac{1-p_{0}}{p_{0}}. \end{array}$$ (55)

Therefore,

$$\begin{array}{c} Eb=\frac{1-p_{0}}{\lambda \left(1-d\left(0\right)\right)p_{0}}, \end{array}$$ (56)

Finally, substituting (42) to (56) completes the proof of (53).

## 6 Numerical examples

### Optimization problems

Before the numerical examples are given, we can ask, what properties a “good” dropping function should have? Unfortunately, such characterization is very hard to obtain given the current state of the analysis of the M/G/1 system with the dropping function.

First of all, there is no way to tell that one dropping function is better than another in general. In fact, one dropping function can be better than another only with respect to a particular cost function. Such cost function may depend, for instance, on the average queue size EX and the loss ratio *L*, but have variable forms, e.g. $C=EX^{a}\cdot L^{b}$ or C=aEX+bL. It may depend also on some other variables, e.g. on the standard deviation of the queue size: $C=EX^{a}\cdot L^{b}\cdot DX^{c}$. Finally, it may be parameterized in several different ways. The final choice of the cost function is arbitrary and depends on our needs. Of course, the shape of a “good” dropping function depends on the choice of the cost function.

Now, even assuming that the cost function is given, it is quite hard to characterize a “good” dropping function. For instance, the common sense tells us that *d*(*n*) should be non-decreasing. Intuitively, there is no reason that the dropping probability should increase, when the queue size decreases. However, it is not easy to prove this formally, given some particular form of the cost function.

All of these do not mean that we cannot effectively use dropping functions in practice to solve optimization problems, at least in some domain. To do this, we have to choose first a cost function and parameterize it. It will be explained below, how this function can be parameterized. Then we have to choose arbitrarily a class of dropping functions depending on a parameter. Then we may find the optimal parameter with regard to the chosen cost function. The obtained solution will not be optimal in general, i.e. in the space of all possible dropping functions, but it will be optimal in the chosen class of dropping functions. Finally, we may repeat the procedure using different classes of dropping functions, e.g. of different shapes and convexity, and compare the costs obtained for them.

Herein, we will use a simple cost being a function of the average queue size and the loss ratio:

$$\begin{array}{c} C=w\;EX+(1-w)L, \end{array}$$ (57)

where *w* ∈ (0, 1) is the weight parameter. To set *w*, we have to decide how bad (costfull) it is when the queue size grows by *x*, compared to when the loss ratio grows by *y*. For instance, we may decide that it is equally bad when the queue size grows by 50, as when the loss ratio grows by 1%. In such example, we obtain immediately that *w* should be about 0.0002, as we have 0.0002 ⋅ 50 ≈ (1 − 0.0002) ⋅ 1%.

Note that the decision how we value losses versus the queue size is arbitrary by nature. For instance, in one computer network we may value a short queue size more (e.g. for gaming, VoIP) while in other, a small loss ratio (e.g. for reliable transfer of documents, files, etc.).

When the cost function is properly defined, we may start searching for the optimal dropping function in some class. The first class used herein is the following:

$$\begin{array}{c} d_{1}\left(n\right)=\{\begin{array}{c} 1-e^{-\frac{n-v}{10}},\;\;\;\text{if}\;\;\;n>v,\\ 0,\;\;\;\text{otherwise}. \end{array} \end{array}$$ (58)

**Fig 1:**
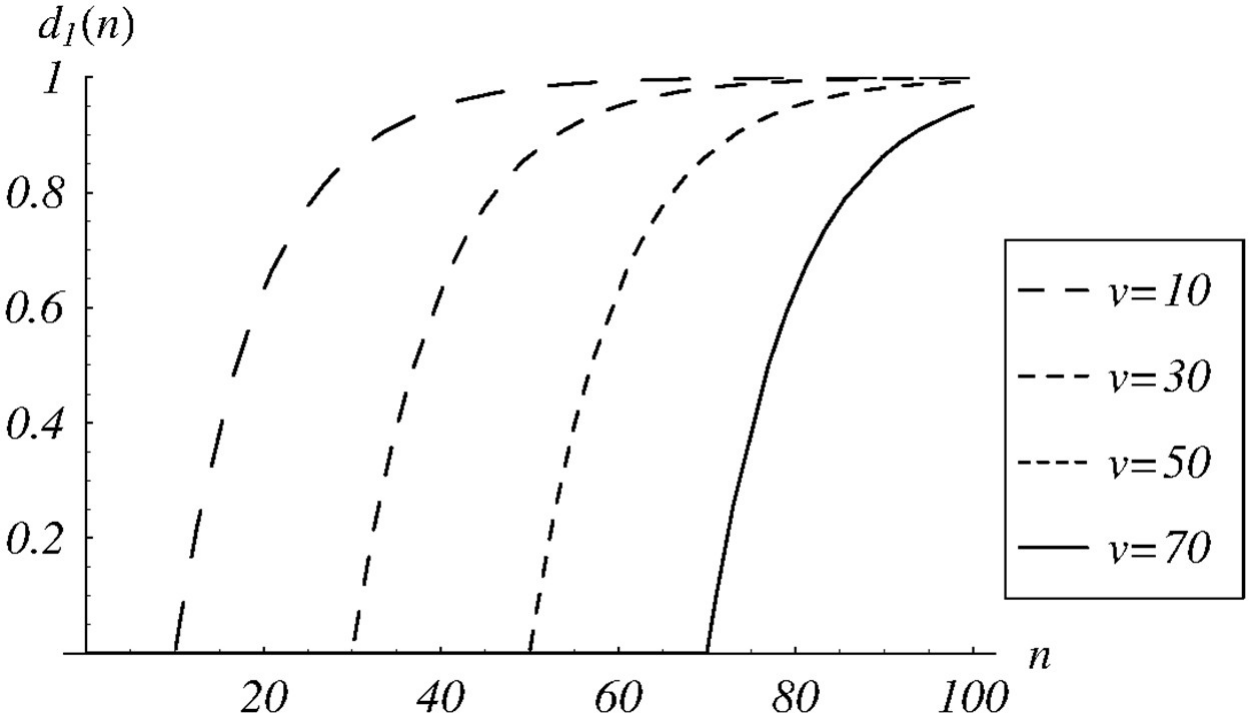
The dropping function *d*_1_(*n*) for different values of the shift parameter *v*.

It depends on shift parameter *v*. A few sample functions from this class are depicted in Fig 1.

We assume that the weight parameter is set to *w* = 0.0002, the system is critically loaded, *ρ* = 1, the service time is constant, and the queue is stable, which follows from Theorem 1.

**Fig 2:**
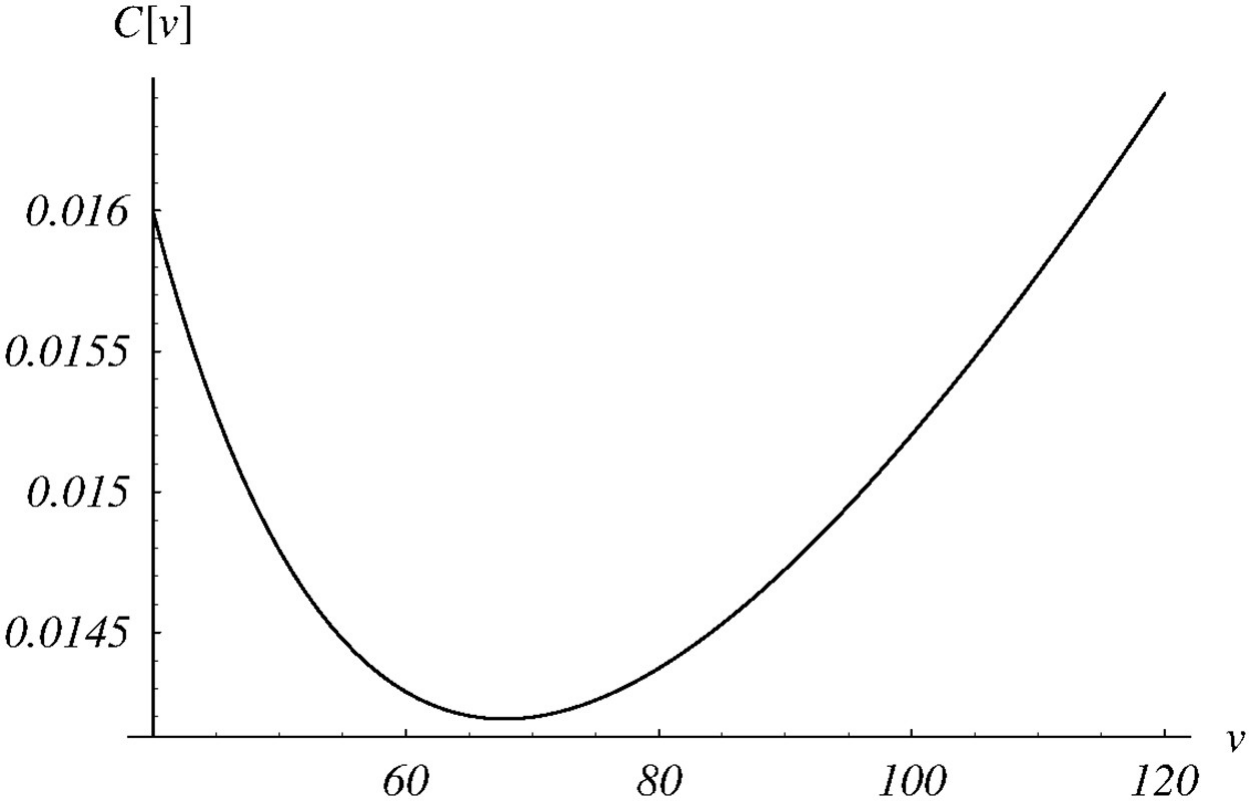
The cost function versus the shift parameter *v* for the class *d*_1_(*n*).

In Fig 2, the dependence of cost (57) on the parameter *v* is depicted for *v* ∈ (0, 120). The obtained numerically minimal cost is reached for *v* = 67.9, which gives the optimal dropping function from the class *d*_1_. Detailed performance characteristics for the queueing system with the dropping function *d*_1_ and *v* = 67.9 are also presented in the fourth column of Table 1.

**Table 1:** System performance for different dropping functions and their parameterizations. In every case, the best trade-off between EX and *L* was achieved using weight *w* from the first row.

	*d*_1_ *v* = 97.9 *w* = 0.0001	*d*_1_, *v* = 28.8 *w* = 0.001	*d*_1_, *v* = 7.1 *w* = 0.01	*d*_1_, *v* = 67.9 *w* = 0.0002	*d*_2_, *u* = 26.6 *w* = 0.0002	*d*_3_, *z* = 0.259 *w* = 0.0002
avg. queue, EX	50.65	16.06	5.28	35.64	34.86	36.58
stddev., DX	29.20	9.37	3.47	20.56	20.16	21.20
loss ratio, *L*	0.496%	1.58%	5.01%	0.707%	0.723%	0.690%
busy period, Eb	200.5	62.2	18.9	140.5	137.3	143.9
cost function, *C*	0.0100	0.032	0.102	0.0142	0.0142	0.0142

**Fig 3:**
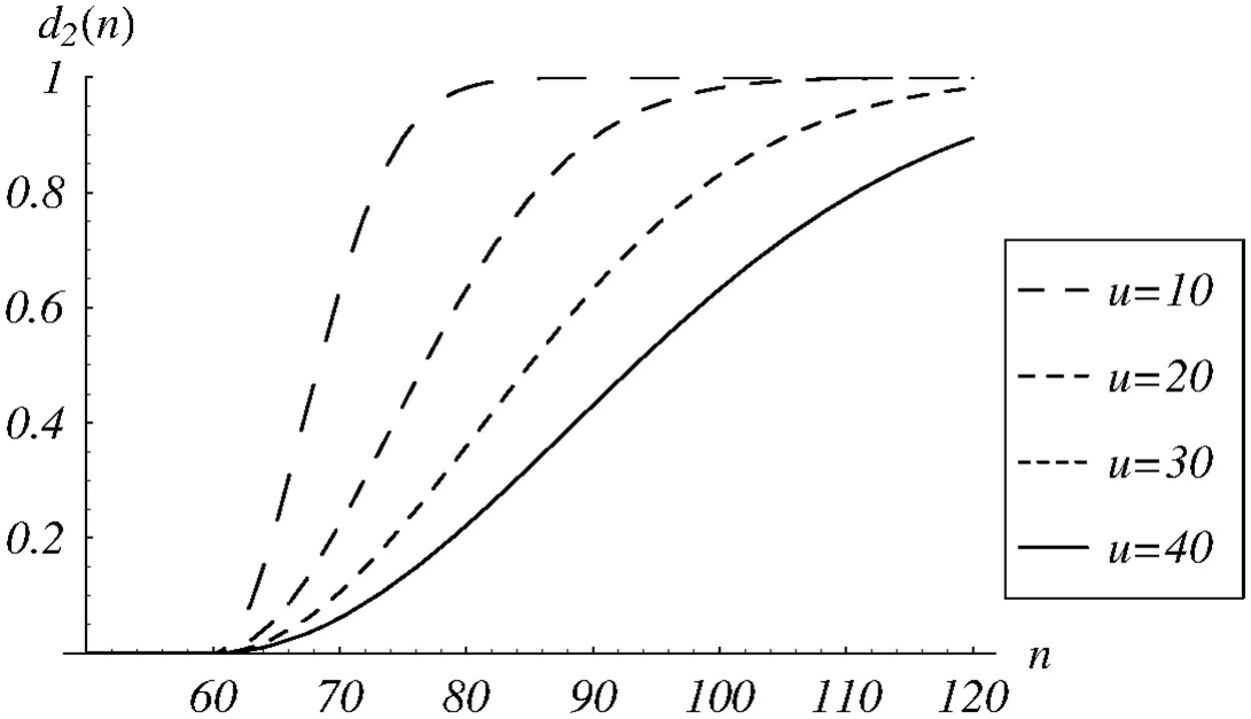
The dropping function *d*_2_(*n*) for different values of the shape parameter *u*.

Obviously, we may search for the optimal solution in other classes of dropping functions, for example in:

$$\begin{array}{c} d_{2}\left(n\right)=\{\begin{array}{c} 1-e^{-(\frac{n-60}{u}{)}^{2}},\;\;\;\text{if}\;\;\;n>60,\\ 0,\;\;\;\text{otherwise}. \end{array} \end{array}$$ (59)

where *u* is now the shape parameter. A few functions from this class are depicted in Fig 3.

**Fig 4:**
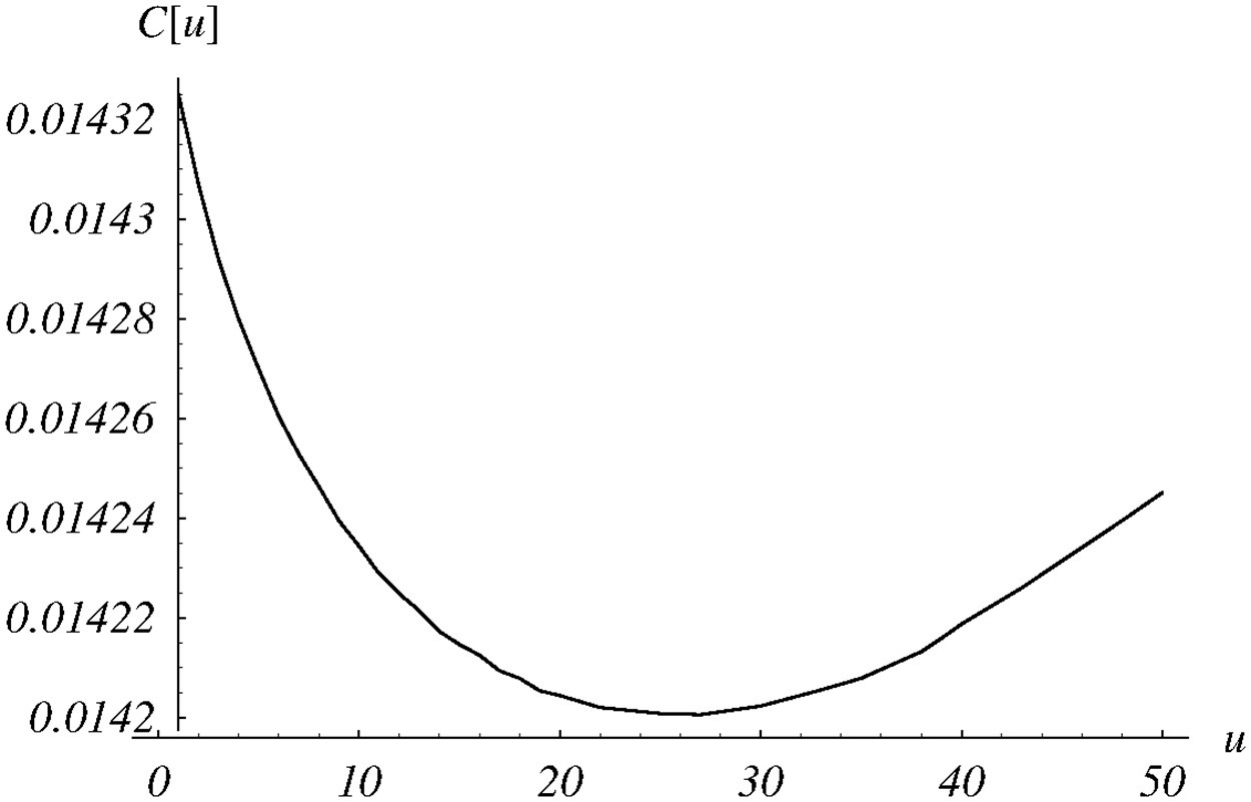
The cost function versus the shape parameter *u* for the class *d*_2_(*n*).

The resulting dependence of cost (57) on *u* ∈ (0, 50) is presented in Fig 4. The obtained numerically optimal parameter value is *u* = 26.6. The performance of the queue for this value of *u* is also summarized in the last but one column of Table 1.

**Fig 5:**
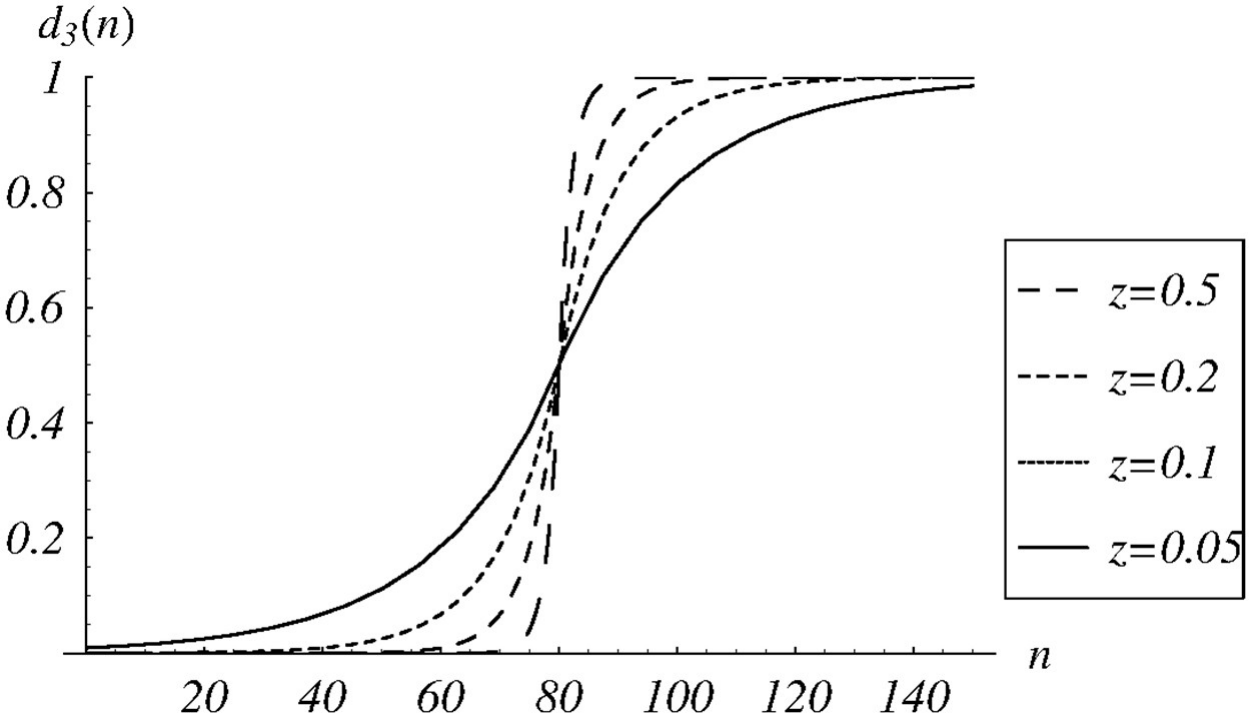
The dropping function *d*_3_(*n*) for different values of the shape parameter *z*.

The third considered class of dropping functions is the following:

$$\begin{array}{c} d_{3}\left(n\right)=\frac{1}{2}+\frac{1}{2}\text{sgn}(z(n-80))\left(1-e^{-|z(n-80)|}\right), \end{array}$$ (60)

where sgn(*x*) denotes the sign of *x*, and *z* is another shape parameter. A few functions from the class *d*_3_, for different values of *z*, are shown in Fig 5.

**Fig 6:**
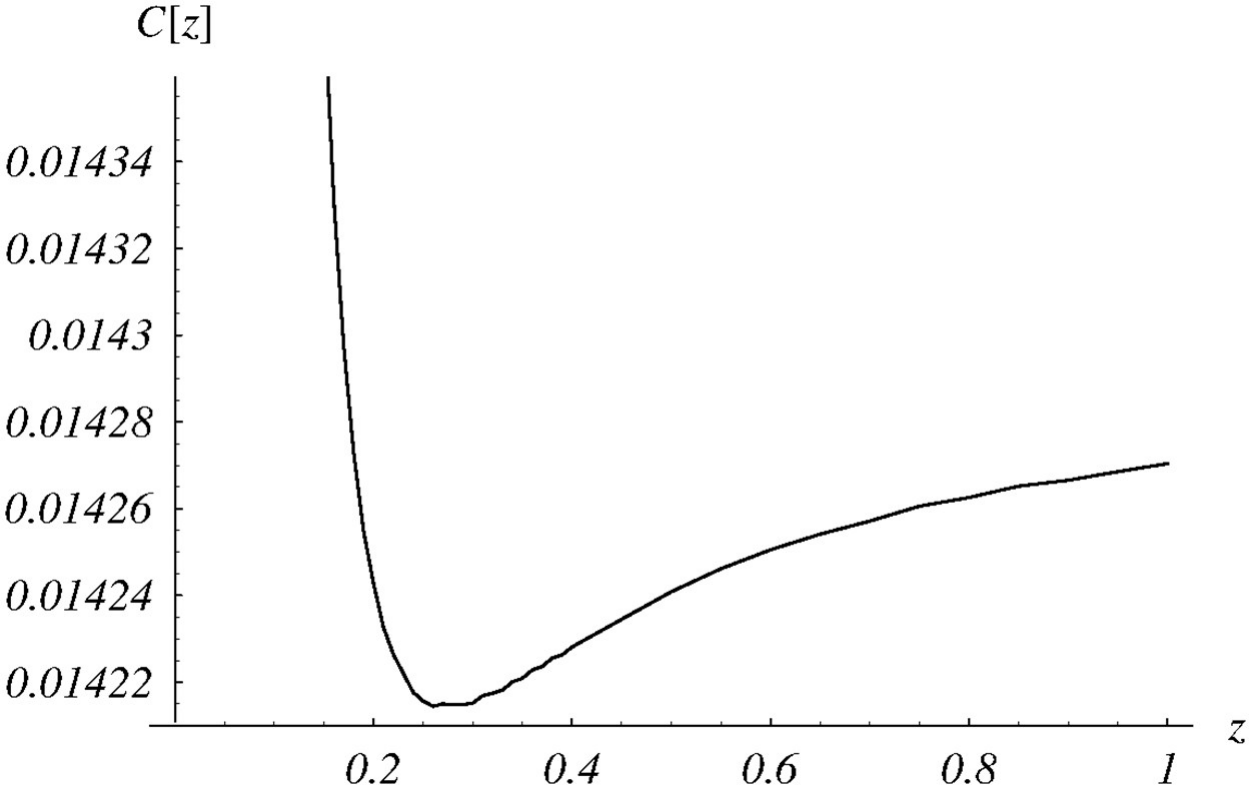
The cost function versus the shape parameter *z* for the class *d*_3_(*n*).

In Fig 6, dependence of cost (57) on *z* ∈ (0, 1) is depicted. The obtained numerically minimum is reached for *z* = 0.259. Detailed performance for this value of *z* is also presented in the last column of Table 1.

Finally, performance characteristics obtained for six different system configurations are gathered in Table 1. In the first three columns, the class *d*_1_ was used to achieve different trade-offs between the queue size and the loss ratio. Namely, the weight parameter *w* varied from 0.0001 to 0.01, resulting in different optimal values of the parameter *v* of the function *d*_1_. As was to be expected, the weight has a deep impact on the queue size, the loss ratio and the optimal cost—they vary significantly in the first three columns of Table 1. On the other hand, in the last three columns, a common weight was used, but with three different classes of dropping functions. As we can see, the optimal results are close to each other. Obviously, this would not happen for any parameter-dependent class of dropping functions. The final, optimal cost depends on the optimization potential of the particular class of functions, and can be higher (or, maybe, lower) than achieved 0.0142. Close to each other results obtained for *d*_1_, *d*_2_ and *d*_3_ classes mean that these classes have similar potential to solve the chosen optimization problem.

### Dependence on the service time distribution

The theoretical results presented herein differ from [10] in the distribution of the service time. Therefore, it is reasonable to ask, how the exponential service time, assumed in [10], influences the performance of the system.

From the classic theory of the M/G/1 system it follows that the average queue size should depend strongly on the standard deviation of the service time, *D*(*F*). (It is rather small in the case of the exponential distribution.) We will study now this dependence.

To accomplish this, the two-point service time distribution is used, with values {0.1, *M*}, and probabilities $p_{0.1}=\frac{M-1}{M-0.1}$ and $p_{M}=\frac{0.9}{M-0.1}$, respectively. As it can be easily checked, the average value of this distribution is 1 for every *M* > 1. However, by manipulating *M* in interval (1, ∞), we can obtain an arbitrary large standard deviation. We assume that the arrival rate is 1, so that the system load is 1 for every value of the parameter *M*. This allows us to study the bare impact of the standard deviation of the service time on the system performance.

**Fig 7:**
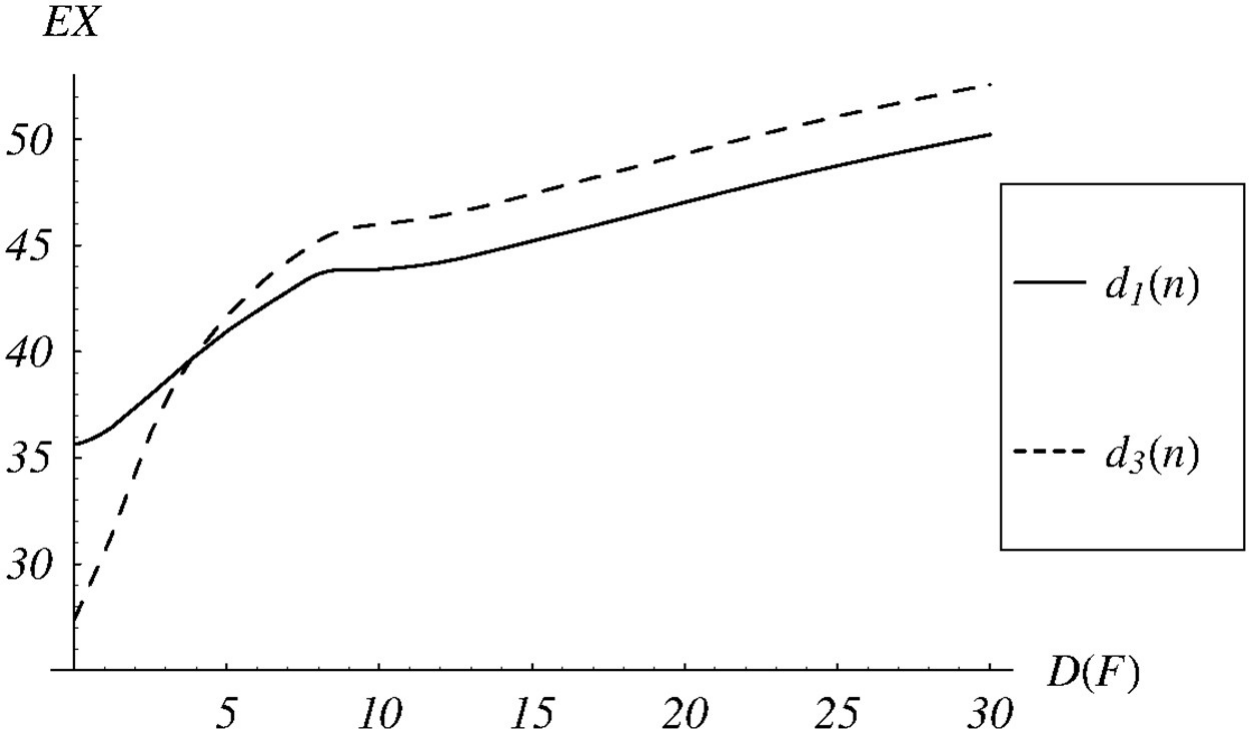
The average queue size vs the standard deviation of the service time for dropping functions *d*_1_ and *d*_3_.

The resulting dependence of the average queue size on the standard deviation of the service time is depicted in Fig 7 for dropping functions *d*_1_ and *d*_3_ with parameters *v* = 67.9 and *z* = 0.1, respectively. As we can see, the dependence has rather irregular form in both cases. Moreover, the average queue size varies significantly. For *D*(*F*) ∈ (0, 30) and *d*_1_, it varies from 35.6 to 50.2. For *d*_3_, it varies from 27.4 to 52.6.

We computed also the average queue size in the case of the exponential service time. For the dropping function *d*_1_ it equals 36.0, while for *d*_3_ it equals 30.3. Comparing these numbers with intervals mentioned in the previous paragraph, we can conclude that the standard deviation of the service time has a deep impact on the average queue size.

**Fig 8:**
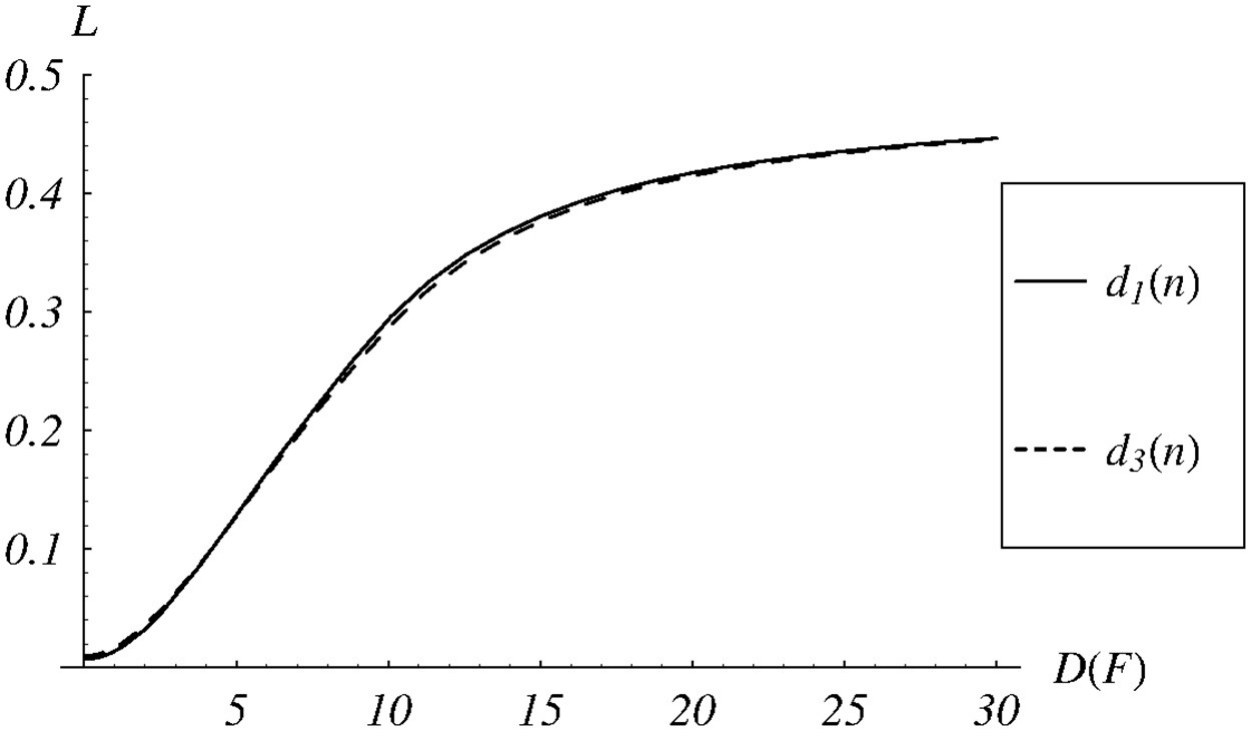
The loss ratio vs the standard deviation of the service time for dropping functions *d*_1_ and *d*_3_.

In Fig 8, the dependence of the loss ratio on the standard deviation of the service time is shown. This time the shapes of both curves are almost identical, but the values vary even more than in the case of the queue size. For both dropping functions, the loss ratio varies from about 1%, up to 44% (!). For comparison, the loss ratio for *d*_1_ and the exponential service time is only 1.4%, while for *d*_3_, it is only 1.7%.

Summarizing, the model with the exponential service time may produce a large error when the real distribution is far from exponential.

### Dependence on the load

In the final set of examples, we check the sensitivity of the average queue size and the loss ratio on the load offered to the system. In the calculations, the variable load in interval (0, 2) is obtained by manipulating the arrival rate. The dropping function *d*_1_ is used with three different parameters: *v* = 5, *v* = 30 and *v* = 70.

**Fig 9:**
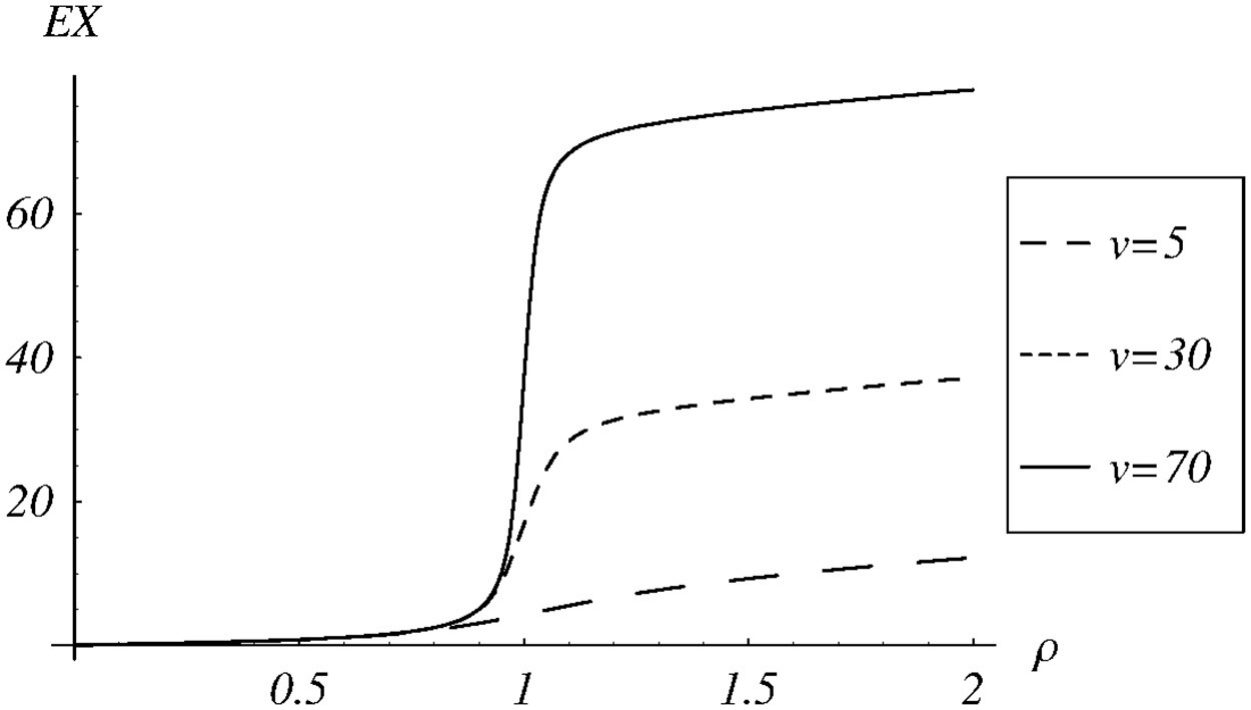
The average queue size vs the system load for the dropping function *d*_1_ with different values of the parameter *v*.

The resulting dependence of the average queue size on the system load is depicted in Fig 9. As we can see, the plot depends significantly on the parameterization of the dropping function, especially for *ρ* > 1. Moreover, it can be observed that the dependence is convex for *ρ* < 1 and concave for *ρ* > 1.

**Fig 10:**
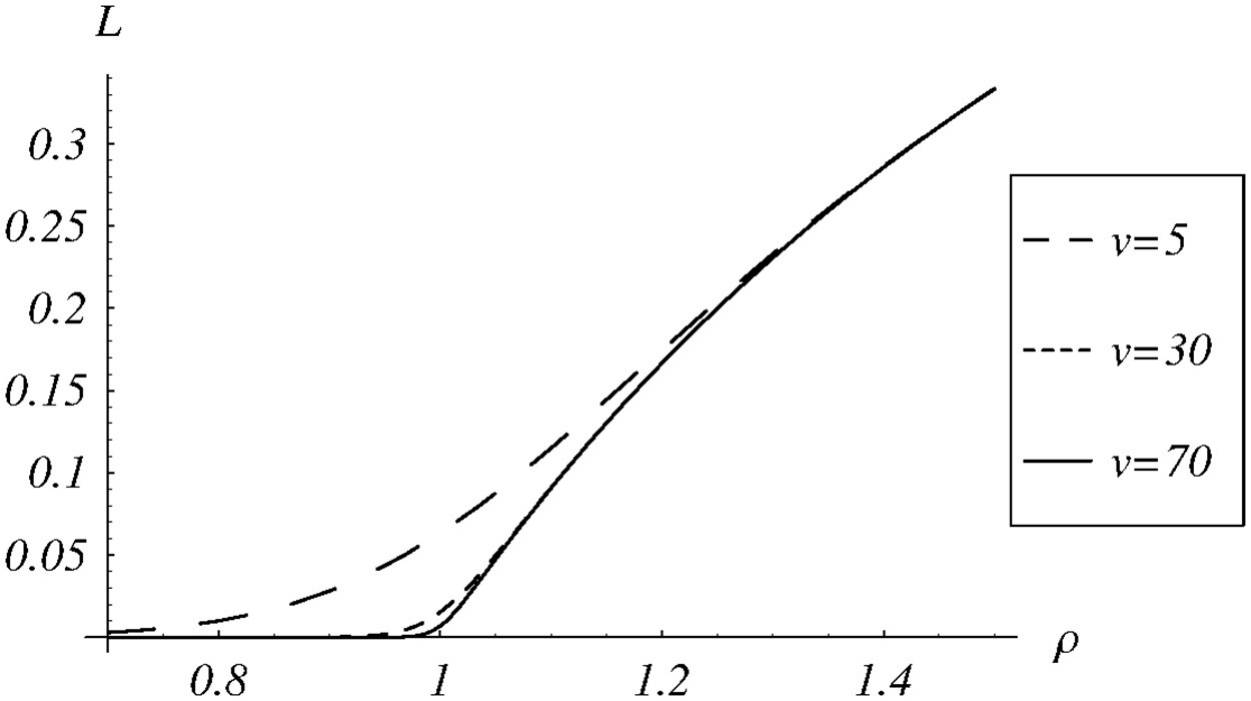
The loss ratio vs the system load for the dropping function *d*_1_ with different values of the parameter *v*.

The dependence of the loss ratio on the system load is depicted in Fig 10. As previously, the shape depends on the parameterization of the dropping function, but less profoundly than in the case of the queue size. As regards convexity, the inflection point is not *ρ* = 1 anymore and its location depends on the parameterization of the dropping function.

## 7 Conclusions

We presented an analysis of the classic M/G/1 queueing model, but with an additional dropping mechanism, based on the dropping function. The study is motivated by the application of the model in networking, namely active queue management in Internet routers. It can be also applied in different areas of computer science, engineering and economics.

We first proved a more general stability condition than the classic *ρ* < 1, which is not useful in the considered model, as far too restrictive. Then, we derived the formulas for the queue length distribution, the loss ratio and the mean duration of the busy period. Finally, we presented numerical results. They were aimed at the optimization of the shape of the dropping function with regard to the combined cost of the queue size and loss ratio, and at demonstrating the dependence of the system performance on the load and the standard deviation of the service time.

There are several possible directions of future work.

Firstly, it would be solving the classic G/M/1 queue with the dropping function, i.e. the system with general renewal arrivals, exponential service, infinite buffer and the dropping function.

Secondly, it would be proving that the dropping function optimal with regard to cost (57), or some other cost, must have some properties, e.g. must be non-decreasing.

Thirdly, it would be proving that the dependence of the average queue size on *ρ* is convex for *ρ* < 1 and concave for *ρ* > 1, as observed in Fig 9.
